# Vaccination to Reduce Antimicrobial Resistance Burden—Data Gaps and Future Research

**DOI:** 10.1093/cid/ciad562

**Published:** 2023-12-20

**Authors:** Birkneh Tilahun Tadesse, Karen H Keddy, Natasha Y Rickett, Aidai Zhusupbekova, Nimesh Poudyal, Trevor Lawley, Majdi Osman, Gordon Dougan, Jerome H Kim, Jung-Seok Lee, Hyon Jin Jeon, Florian Marks

**Affiliations:** International Vaccine Institute, Seoul, Republic of Korea; Division of Clinical Pharmacology, Department of Laboratory Medicine, Karolinska Institutet, Karolinska University Hospital Huddinge, Stockholm, Sweden; Center for Innovative Drug Development and Therapeutic Trials for Africa, College of Health Sciences, Addis Ababa University, Addis Ababa, Ethiopia; Independent Consultant, Johannesburg, South Africa; International Vaccine Institute, Seoul, Republic of Korea; International Vaccine Institute, Seoul, Republic of Korea; International Vaccine Institute, Seoul, Republic of Korea; Wellcome Sanger Institute and Microbiotica, Cambridge, United Kingdom; Cambridge Institute of Therapeutic Immunology and Infectious Disease, University of Cambridge School of Clinical Medicine, Cambridge, United Kingdom; Cambridge Institute of Therapeutic Immunology and Infectious Disease, University of Cambridge School of Clinical Medicine, Cambridge, United Kingdom; International Vaccine Institute, Seoul, Republic of Korea; Seoul National University, College of Natural Sciences, Seoul, Republic of Korea; International Vaccine Institute, Seoul, Republic of Korea; International Vaccine Institute, Seoul, Republic of Korea; Cambridge Institute of Therapeutic Immunology and Infectious Disease, University of Cambridge School of Clinical Medicine, Cambridge, United Kingdom; Madagascar Institute for Vaccine Research, University of Antananarivo, Antananarivo, Madagascar; International Vaccine Institute, Seoul, Republic of Korea; Cambridge Institute of Therapeutic Immunology and Infectious Disease, University of Cambridge School of Clinical Medicine, Cambridge, United Kingdom; Madagascar Institute for Vaccine Research, University of Antananarivo, Antananarivo, Madagascar; Heidelberg Institute of Global Health, University of Heidelberg, Heidelberg, Germany

**Keywords:** antimicrobial resistance (AMR), data gaps, vaccination, indirect effect, study design

## Abstract

Antimicrobial resistance (AMR) poses an immediate danger to global health. If unaddressed, the current upsurge in AMR threatens to reverse the achievements in reducing the infectious disease–associated mortality and morbidity associated with antimicrobial treatment. Consequently, there is an urgent need for strategies to prevent or slow the progress of AMR. Vaccines potentially contribute both directly and indirectly to combating AMR. Modeling studies have indicated significant gains from vaccination in reducing AMR burdens for specific pathogens, reducing mortality/morbidity, and economic loss. However, quantifying the real impact of vaccines in these reductions is challenging because many of the study designs used to evaluate the contribution of vaccination programs are affected by significant background confounding, and potential selection and information bias. Here, we discuss challenges in assessing vaccine impact to reduce AMR burdens and suggest potential approaches for vaccine impact evaluation nested in vaccine trials.

## Teaser

Antimicrobial resistance (AMR) represents a significant global health threat. Vaccination can decrease AMR through reduction of antimicrobial use and prevention of secondary infections caused by antimicrobial-resistant pathogens. Better studies are needed to quantify vaccine impacts on AMR.

In 2015, the 68th World Health Assembly stated that the impact of antimicrobial resistance (AMR) on global health and economies was “… a heavy and growing burden on high-, middle- and low-income countries, requiring urgent action at national, regional and global levels, particularly in view of the limited development of new antimicrobial agents” [1]. That year, the World Health Organization (WHO) initiated the Global Antimicrobial Resistance and Use Surveillance System, a reporting system that collates information on AMR and antimicrobial consumption (AMC) in humans for selected pathogens [2]. By 2021, 111 of 216 countries, territories, and areas were enrolled, including much of sub-Saharan Africa (sSA) and South Asia. However, limited antimicrobial susceptibility testing (AST) and data collection coverage in low- and middle-income countries (LMICs) has hindered accurate calculation of AMR burden [2].

Despite active programs to combat AMR, its rates between 2017 and 2020 increased by more than 15% for bacteremias, meropenem, and third-generation cephalosporin resistance in *Escherichia coli*, ciprofloxacin resistance in *Salmonella* spp., and azithromycin resistance in *Neisseria gonorrhoeae* [2]. An estimated 3.6 million deaths in 2019 were associated with AMR infections caused by six pathogens: *Staphylococcus aureus, E. coli, Streptococcus pneumoniae, Klebsiella pneumoniae, Acinetobacter baumannii, and Pseudomonas aeruginosa* [3]. LMICs bore a disproportionate burden of this mortality because of limited AST for targeted antimicrobial treatment [2]. In sSA and South Asia, respectively, the disability adjusted life-years (DALYs) were 6,144, and 3,318 [3]. There were 1.6 million deaths from tuberculosis in 2021, of those 191,000 were caused by drug-resistant M. tuberculosis during the COVID-19 pandemic, an increase compared with previous years that particularly affected Southeast Asia and sSA [4]. Nine of the 10 countries with the highest multidrug-resistant (MDR)-TB/rifampicin-resistant TB burden are in sSA or Southeast Asia (China, the Democratic Republic of the Congo, India, Indonesia, Nigeria, Pakistan, the Philippines, South Africa, and Viet Nam) [4]. In 2019, *S. pneumoniae* contributed to 16% of AMR-attributable deaths in sSA, compared with 7% in high-income countries [3].

**Figure 1.**
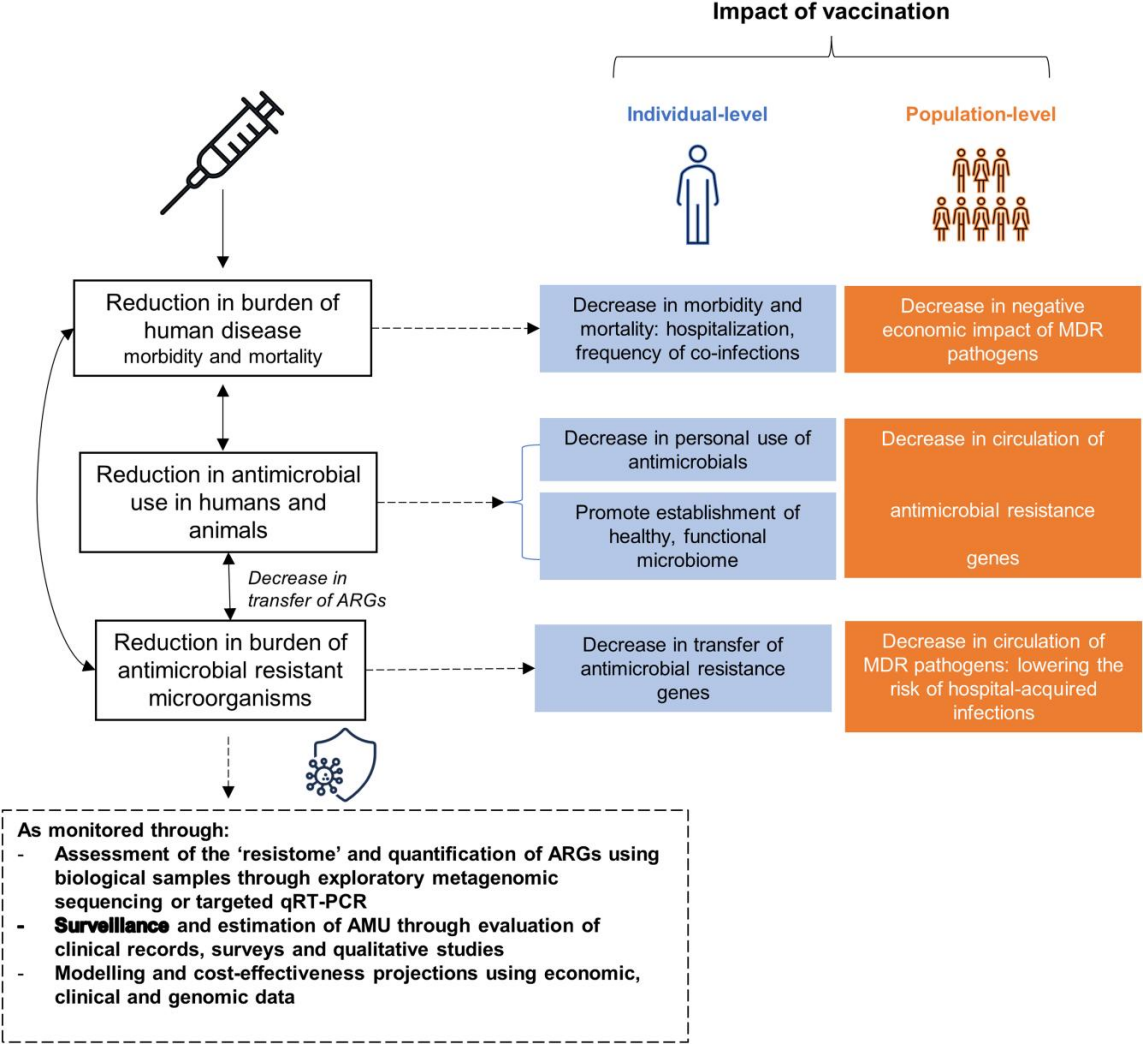
Understanding how vaccines can mitigate antimicrobial resistance (AMR) at the individual and population level. Abbreviations: AMU, antimicrobial use; ARG, antimicrobial resistance gene; MDR, multidrug resistance; qTR-PCR, quantitatiove real-time polymerase chain reaction.

A significant knowledge gap exists regarding the economic burdens of AMR. A rapid methodological review on the economic cost of antibiotic resistance reported that most studies (91 of 110) were carried out in high-income countries, with 48 of 91 studies being conducted in the United States [5]. Thus, LMICs are disproportionately underrepresented in existing studies on the economic burden of AMR. Here, we review how vaccines might directly and indirectly reduce the burden of AMR and discuss methods by which this impact can be quantified and standardized in future and ongoing clinical trials (Figure 1).

## EVIDENCE ON THE IMPACT OF BACTERIAL AND VIRAL VACCINES IN REDUCING AMR

Effective vaccines for common community-acquired and hospital-associated infections (HAIs) (*E. coli*, *Haemophilus influenzae*, *Mycobacterium tuberculosis*, *N. gonorrhoeae*, *Salmonella enterica* serovar Typhi, *Salmonella enterica* serovar Paratyphi, invasive nontyphoidal *Salmonella*, *Shigella* spp., *S. pneumoniae*, *Streptococcus pyogenes* (Group A Streptococcus, *S. aureus*, *K. pneumoniae*, *P. aeruginosa*, *A. baumannii*, *Enterococcus faecium*) would avert 490 000 deaths and 28 million DALYs associated with AMR globally. The greatest impacts would be in Africa (164 000 deaths and 12 million DALYs) and Southeast Asia (53 000 deaths and 2 million DALYs) [6].

Decreasing the burden of disease due to susceptible and resistant pathogens should decrease antimicrobial use (AMU), potentially increasing the proportion of susceptible organisms isolated. Postintroduction evaluation studies demonstrated nonsusceptibility in *S. pneumoniae* isolates to first-line antimicrobials decreased globally following the introduction of pneumococcal conjugate vaccine (PCV), with a 12% decrease in non-susceptibility to penicillin [7]. For example, following the introduction of PCV7 then PCV13 (7- and 13-valent PCV) in a cohort of patients aged 6 months to 3 years from 2006 to 2016, penicillin resistance in all pneumococcal serotypes decreased. Resistance increased thereafter as novel *S. pneumoniae* serotypes replaced vaccine strains [8]. A more effective pneumococcal vaccine would have more impact on mortality, averting 120 000 deaths [6].

Comparable data for *H. influenzae* type B (Hib) conjugate vaccine (Hib-CV) are scarce, particularly for LMICs. The global estimate for Hib disease in children younger than age 5 years was ∼340 000 severe episodes in 2015, with 30 000 deaths and excessive burdens in Africa and Southeast Asia, but these estimates excluded AMR Hib data [9]. A Portuguese study reported a decrease in *β*-lactamase-mediated ampicillin-resistant Hib from 27% to 10% between 2001 and 2010 following Hib-CV rollout [10]. A South African study, however, showed a 71% decrease in reported invasive Hib disease, but increased MDR, including to ampicillin, from 2% to 19% between 2000 and 2004 [11]. Similarly, in Japan, ampicillin resistance associated with Hib meningitis increased from 6% in 2,000% to 35% in 2004 [12] and to 70% in 2011 [13], following Hib-CV introduction in 2008, despite a 93% reduction in invasive Hib disease [14]. Increased burdens of invasive nontypeable *H. influenzae* strains or non-Hib capsular serotypes have been described in children, reversing the initial reduction in *H. influenzae* disease burdens [10, 15]. AMR was associated with these replacement strains [10], which may also drive increased MDR in Hib disease.

Extremely drug-resistant (XDR) *S.* Typhi caused a large outbreak of typhoid fever in Pakistan in 2017–2018, with more than 5,000 documented cases [16]. The resultant rapid rollout of typhoid conjugate vaccine (TCV) enrolled 207 000 children aged 6 months to 10 years [17]. In a cohort of 23 407 children, vaccine effectiveness was 97% against XDR *S*. Typhi and 95% against all culture-confirmed *S.* Typhi [17]. A cholera vaccine trial during an MDR cholera outbreak in Guinea in 2012 [18] was 87% effective in protection against *Vibrio cholerae* O1 [19]. Notably, no fully antimicrobial susceptible isolates of *V. cholerae* O1 from Africa have been isolated since 2000 [20].

The COVID-19 pandemic had a well-documented potentiating effect on AMR. Besides increased MDR bacterial co-infections [21, 22], AMU increased significantly in the early stages of the pandemic, despite limited evidence of bacterial or fungal co-infection in most patients [23, 24]. This became a global phenomenon as the pandemic spread [25–28]. Nonetheless, the pandemic also demonstrated the substantial versatility of the global medical research community and its ability to develop safe and effective vaccines within short timeframes [29, 30]. This underscores the impact of vaccines on averting diseases and showcases that vaccination now represents a viable and cogent tool in reducing the burden of AMR.

The direct and indirect impact of vaccines on AMR has been incompletely explored. Furthermore, accounting for the effects of other public health interventions on AMR requires properly designed and controlled studies. Clinical trials for vaccines at different stages of clinical development provide a unique opportunity to incorporate AMR/AMU-related secondary and exploratory endpoints.

## MECHANISMS BY WHICH VACCINES COULD CONTRIBUTE TO REDUCTIONS IN AMR

### Vaccination to Reduce AMU/AMC

AMU may increase resistance, selecting for it in the target pathogen and other pathogenic and nonpathogenic organisms, providing a proxy for the risk of AMR development. Horizontal gene transfer of antimicrobial resistance genes (ARGs) occurs via mobile genetic elements, including plasmids, between bacteria [31]. Development of AMR may be driven by the use of antimicrobials in animals and environmental exposures, as much as individual and large-scale use in humans. Although unequivocal evidence is scant [32], there are data suggesting that AMR may be transmitted to humans from food animals treated with antimicrobials for growth promotion [33]. The gut microbiome may harbor ARGs that can be transmitted to pathogens [34] and between animals and humans [35]. Analysis of the gut microbiome of food animals in China revealed that mobile genetic elements harboring ARGs were shared between human and gut microbiomes, and additionally by human pathogens, including *E. coli*, *Streptococcus agalactiae* (Group B *Streptococcus*), and *E. faecium* [35]. ARGs encoding MDR in *Campylobacter*, a common foodborne pathogen, have been shown to be transmitted between livestock, sewage, and humans [36]. An extensive epidemiological analysis of human cases of *Campylobacter* in the Netherlands complemented by whole-genome sequencing of *Campylobacter* isolates from humans, companion animals, food animals, and water (environmental samples) indicated acquisition of human campylobacteriosis from multiple sources [37]. Sewage contamination of coastal waters has resulted in the transmission of AMR in *Campylobacter* and nontyphoidal *Salmonella* to seabirds [38], which may contaminate other water sources [37]. Trials with azithromycin in Africa to improve childhood mortality (a single dose of azithromycin given twice yearly to children from aged 1 to 59 months) [39], were associated with increased resistance to macrolides in nasopharyngeal *S. pneumoniae* after 2 years [40]. Characterization of ARGs from rectal swabs showed a 7-fold increase in resistance determinants to macrolides and a 2-fold increase to other antimicrobials, including *β*-lactams, in a subset of study subjects within 3 years [41]. Vaccination may decrease the occurrence of co-infections or secondary infections, impacting AMU [42], and thereby further decreasing other AMR pathogen-associated disease burdens, besides the burden attributable to the pathogen targeted by the vaccine (Table 1).

**Table 1.** Selected Examples of Direct and Indirect Impacts of Existing or Soon-to-be-Developed Vaccines on Antimicrobial Resistance (AMR) and Antimicrobial Use (AMU)

Target Organism(s) and Vaccine	Major Clinical Syndromes	Potential Impact on AMR and AMU in Associated Primary Infections, Co-infections, and Secondary Infections
Bacterial vaccines		
*Streptococcus pneumoniae* Pneumococcal conjugate vaccine (PCV)—various formulations	Pneumonia, acute *otitis media*, bacterial meningitis	A 2-year randomized double-blind clinical trial evaluating PCV7 against PCV13 in >1,800 Israeli infants established PCV13 was significantly more effective in reducing the prevalence of nasopharyngeal carriage from all MDR *S. pneumoniae* serotypes (≥3 antimicrobials), including nonvaccine serotypes (odds ratio, 0.83; 95% confidence interval, .68–1.01) [43]
*Haemophilus influenzae* type b (Hib) and nontypeable *Haemophilus influenzae* Hib conjugate vaccine (Hib-CV) *Haemophilus influenzae* protein D conjugate vaccine (PHiD-CV10)	Pneumonia, acute *otitis media*, meningitis	A significant decrease in Hib in Portuguese children, from 81% to 13%, (*P* < .001), was associated with a decrease in β-lactamase–producing strains [10] A Finnish vaccine trial examined AMU in >45,000 children randomized to receive a 10-valent pneumococcal nontypeable *Haemophilus influenzae* protein D conjugate vaccine (PHiD-CV10). AMU decreased among PHiD-CV10 recipients compared with controls [44, 45]. A calculated decrease of 12,000 antimicrobial purchases per year in children younger than age 2 y in Finland was observed in a birth cohort of 60,000 children when PHiD-CV10 was administered [46], whereas multidrug resistance (MDR) decreased from 22% to 6% (*P* < .001) [47]
*Salmonella enterica* serotype Typhi Typhoid conjugate vaccine (TCV)	Enteric fever	A decrease in MDR *S.* Typhi infections was calculated at a global level, although the proportion of MDR *S.* Typhi would remain constant [48]
*Mycobacterium tuberculosis* Bacillus Calmette-Guérin (BCG) Improved vaccine for tuberculosis	Pulmonary and extrapulmonary tuberculosis (TB)	An effective postexposure vaccine would avert 10% of TB cases and 7% of deaths because of rifampicin-resistant TB (RR-TB), multidrug-resistant TB (MDR-TB), and extremely drug resistant TB (XDR-TB) [49] Selective pressures of antimicrobial use for MDR-TB in other pathogens would be averted, such as fluoroquinolone resistance in *S. pneumoniae* [50] and *C. difficile* [51] BCG vaccination has been shown to reduce all-cause mortality by half in children aged younger than age 5 y [52]
*Neisseria gonorrhoeae*	Sexually transmitted infections (STIs), spontaneous abortion, stillbirth, preterm birth, low birth weight and perinatal morbidity and mortality	Decrease in the number of AMR gonorrhea cases, which may emerge within 3–4 years of the introduction of AMU in revised treatment guidelines [53], which could drive AMR in other sexually acquired pathogens, such as azithromycin-resistant *Shigella* [54]
Nontyphoidal *Salmonella* (NTS)	Diarrhea, dysentery, invasive salmonellosis (iNTS disease)	In sSA, iNTS may be as high as 383 per 100,000 population, and 48% of iNTS isolates were MDR [55]. In the United States, infections from AMR NTS are associated with increased risk of iNTS (adjusted OR, 1.6) and hospitalization (adjusted OR, 3.1) compared with fully susceptible NTS [56]
*Klebsiella pneumoniae*	Hospital-associated infections (HAI)	MDR *K. pneumoniae* contributed to 20% of the deaths attributable to AMR [3]. An effective vaccine would both decrease this burden as well as reduce selective pressures of AMU on the development of *C. difficile* infection [57–59] Emergence of a novel extended-spectrum *β*-lactamase enzyme in *K. pneumoniae* HAI in South Africa in the mid-1990s (TEM-63) [60] was later followed by the identification of TEM-63 in HAI *Salmonella* infections in the early 2000s [61], and community-acquired *Vibrio cholerae* O1 in 2008 [62]
Viral vaccines		
Influenza Influenza A, B (depending on circulating serotypes)	Seasonal influenza	Excessive mortality during the influenza season is associated with secondary bacterial infections, particularly *S. pneumoniae*, followed by *Staphylococcus aureus*, but significantly fewer antibiotic courses are reported in vaccinated children (*P* < .0001) [63]. Temporal variations in *C. difficile* incidence followed temporal variations in influenza infections (*P* = .043) [64]. In the United States, between 2010 and 2017, a 10% increase in vaccination rates for influenza was associated with a 6.5% decrease in antibiotic prescriptions, particularly in children (6% reduction between ages 0 and 18 y) and older patients (5.2% reduction in patients >65>y) [65]
SARS-CoV-2 (COVID-19 vaccines—multiple formulations)	COVID-19	During the pandemic, the proportion of bacterial or fungal co-infection in patients with confirmed SARS-CoV-2 ranged from 3% to 100%, with AMR to at least 1 antimicrobial [21]. Additionally, antimicrobial usage increased by an estimated 68% to 75% in 2 separate studies [27, 28]
Rubeola virus Measles vaccine, measles mumps rubella (MMR)	Measles	Between 1990 and 2008, the measles vaccine reduced all-cause mortality in children younger than age 5 y by 28% [66]. Within the first 2 y of life, all-cause mortality was halved [52]
Rotavirus vaccine	Diarrhea	The 5-y age-adjusted cumulative incidence against antibiotics prescribed in eligible children with commercial health insurance, who had been fully vaccinated for Rotavirus compared with those who had received no vaccine, was 0.793, between 2007 and 2018 [67]
Respiratory syncytial virus (RSV)	Bronchiolitis, pneumonia	Immunization of pregnant mothers resulted in the transfer of neutralizing antibody titers to newborn infants, with an associated efficacy of 92% from severe RSV-associated lower respiratory tract illness [68]. RSV infection may enhance MDR *Pseudomonas aeruginosa* colonization in chronic lung disease [69], temporal variations in *C. difficile* incidence followed temporal variations in RSV (*P* = .004) [64]
Human immunodeficiency virus (HIV)	HIV, AIDS	Bacterial and fungal co-infections, including resistant *S. pneumoniae*, iNTS, and MDR-TB, are primarily responsible for excessive mortality in HIV-infected patients [70–75], who often receive antimicrobial prophylaxis
Parasitic vaccines		
*Plasmodium falciparum*	Malaria	A decreased burden of AMR malaria would be expected [76], and of co-infection with AMR invasive salmonellosis in Africa [77], which further drives AMU

Abbreviations: AMR, antimicrobial resistance; AMU, antimicrobial use; COVID-19, coronavirus disease 2019; Hib, *Haemophilus influenzae* type B; SARS-CoV-2, severe acute respiratory syndrome coronavirus 2; TB, tuberculosis.

### Vaccination, AMR, Metabolic Costs, and Role of the Microbiome

The fitness cost of acquiring MDR plasmids is significantly greater than acquiring those with monoresistance (*P* < .05), whereas de novo development of chromosomal resistance is associated with a greater cost than plasmid acquisition [31]. The XDR-*S.* Typhi isolated during the Pakistani typhoid fever outbreak expressed plasmid and chromosomally mediated AMR, which may explain the greater efficacy of TCV against XDR-*S*. Typhi in Pakistan (97%) [17] compared with that in Nepal (79%) [78], Malawi (81%) [79], and a controlled human infection model (52% to 55%) [80]. Globally, PCV reduced the prevalence of penicillin nonsusceptibility in *S. pneumoniae* (PNSP) among PCV serotypes by 16% [7]. In contrast, in Massachusetts, USA, despite reduced nasopharyngeal carriage of PNSP in children who had received PCV13, the proportion of PNSP PCV13 serotypes remained constant [81]. These differences may be explained by initial fitness costs associated with the acquisition of penicillin-binding protein genes in *S. pneumoniae* being offset by the evolution of compensatory mutations, increasing resistance to β-lactam antibiotics [82]. Similarly, *E. coli* that acquired amoxicillin resistance resulted in increased glucose consumption initially followed by reduced salt and pH tolerance, but ultimately no difference in maintenance energy [83]. Fitness costs may, hence, be exploitable in vaccination strategies for certain MDR pathogens.

Changes in the human microbiome, which mediates physiological processes including pathogen transmission, immune system development, and nutritional status, could partially explain the interplay between vaccination and the acquisition, development, and evolution of AMR. The first year of life is critical for the establishment of a healthy, functional microbiome, priming the immune responses to vaccine-preventable infections that disproportionately affect newborns and infants [84]. *Bifidobacterium*, for instance, metabolizes human milk oligosaccharides in the neonatal gut, producing short-chain fatty acids, which promote differentiation of colonic T cells into T-regulatory cells, mediating the production of antigen-specific immunoglobulin A [85, 86].

AMR-containing opportunistic pathogens may also colonize the early-life gut microbiome [85, 87]. Horizontal transfer of ARGs can also be enhanced or attenuated by commensals and pathogens present in the gut microbiome [88].

Manipulating the microbiome may mediate vaccine efficacy and AMR carriage, directing vaccine development and co-interventions to enhance vaccine response. Microbiota-informed dietary recommendations, novel probiotics, or prebiotic adjuvants to promote the growth of gut commensals could stimulate vaccine response and improve vaccine efficacy [89].

## OUTCOME MEASURES FOR STUDIES OF VACCINE IMPACT ON AMR

The TCV cohort study in Pakistan following the typhoid fever outbreak in 2017–2018 noted an impact of the vaccine on the isolation of XDR *S*. Typhi within 2 years of initiating vaccination [17]. Similarly, introduction of PCV in Finland was followed by an observed reduction in penicillin-non-susceptible *Streptococcus pneumoniae* (PNSP) within 5 years [47]. These included large study populations; smaller studies may be underpowered to detect an effect. Evaluating vaccine impacts on AMR and AMU should be vaccine- and pathogen-specific, but this realistically depends on trial design.

A search for currently recruiting, ongoing, and completed studies in ClinicalTrials.gov including vaccine interventions listed more than 1,000 relevant studies. Of these, 119 trials referenced terms related to antimicrobial resistance, usage, or prescription; 16 trials included AMR as a stated primary or secondary outcome in an interventional or observational vaccine trial; 14 of 16 stated monitoring AMR as a secondary outcome; and 2 included AMU as a primary outcome (Supplementary Figure 1; Supplementary Table 1). Study outcomes predominantly focused on AMC or AMU postvaccination (6 and 4, respectively), whereas 5 studies aimed to assess AMR profiles and detect specific resistance genotypes or phenotypes. One study included AMR and AMU as secondary outcomes. The impact of PCV on MDR was the predominant subject of the studies listed.

Standardization will be critical for comparing future studies defining the impact of vaccines on AMR. Currently, studies focus primarily on vaccine safety and efficacy, with impact on AMR being typically included in posttrial analyses. Secondary analyses predominantly emphasize vaccine characteristics including long-term effectiveness of vaccines in the community and durability of protection [90]. This lack of systematically collected AMR data may occasion discrepancies in diagnostic methods for pathogen isolation, differences in strain characterization, loss of critical isolates, and variation in data collection and quality, particularly in multicenter studies. The diversity of analytical methods pre- and posttrial may affect post hoc results. Although the WHO has published guidelines for the clinical evaluation of new vaccines, these focus on immune response, safety, efficacy, and effectiveness [90, 91], rather than pathogen characterization in vaccine-preventable diseases.

Metagenomic sequencing of ARG burdens in communities of pathogenic and nonpathogenic bacteria (the “resistome”) offers further opportunities to gather evidence on the potential of vaccines to control AMR. Clinical trials of microbiome-targeted therapies reduced AMR carriage in individuals (eg, fecal microbiota transplantation) pre- and posttreatment [92]. Evaluating ARG burdens in wastewater could also inform our understanding of the impact of vaccines on AMR at the community level [93, 94]. Such studies would require careful consideration of the inherent limitations in the detection limits of metagenomics, necessitating consensus on optimal methods.

## LEVERAGING VACCINE STUDIES FOR EVALUATION OF VACCINE IMPACT ON AMR

Global stakeholders and funders should encourage a standardized approach for pathogen surveillance of (potentially) vaccine-preventable diseases. Whereas AMU is a key component of disease prevention and management, AST should be performed on the relevant pathogens and AMU should be monitored in infectious disease surveillance. These should be secondary outcomes in study subjects of vaccine trials, along with the impact on AMU and AMR on co-infections or superinfections.

### Randomized Controlled Trials

Phase III/IV trials, if sufficiently powered, provide an opportunity to examine the potential impact of vaccines on AMU and AMR [95]. The Finnish and Israeli randomized controlled trials (RCTs) comparing PCV and Hib-CV vaccine formulations (Table 1) illustrated the large numbers required to establish vaccine impact on AMU and AMR. These studies also demonstrated the timespan needed for follow-up, frequency with which MDR strains may be expected to be isolated, and the specimen type for optimal statistical analysis, when examining nasopharyngeal carriage rather than invasive disease [43, 44, 46]. Future and upcoming RCTs should measure AMU/AMC and AMR in vaccine and control groups, for the primary pathogen and selected Global Antimicrobial Resistance and Use Surveillance System pathogens of interest.

Although current methods to “quantify” AMR content in the microbiome are challenging, this technique will be available in the foreseeable future. Given the relatively long observation times in phase III and IV studies, collecting samples in a subset of participants (ie, stool and nasopharyngeal swabs) may enable the retrospective assessment of the indirect impact of vaccines on AMR.

### Observational Studies

Most data concerning the impact of vaccination on AMR are collected from observational studies that compare AMR data in the pathogen(s) of concern before and after vaccine introduction [7, 45, 47]. Observational studies to define the impact of vaccines on AMR require systematic laboratory surveillance, either of a cohort of vaccinees [17] or at a pathogen-specific level in a population, including active disease surveillance and carriage [10, 96, 97]. For instance, following the PHiD-CV10 RCT trial in Finland, the incidence of AMR in clinical *S. pneumoniae* isolates declined significantly in all age groups, which is indicative of the wider impact of vaccination beyond vaccine recipients [47]. Similar studies have been cited in Table 1 (above), but this list is not exhaustive.

Table 2 summarizes the key research questions regarding vaccine impact on AMR, example(s) of the potential study designs, and some of the limitations/challenges based on the stage of the clinical vaccine trial.

**Table 2.** Evaluation of Vaccine Impact on AMR as Part of Clinical Trials Designed for Vaccine Development

Vaccine Trial Design	AMR-Specific Research Aims That Can Be Addressed as Secondary or Exploratory Objectives	Study Designs To Address AMR-Specific Research Objectives	Limitations and Other Considerations
Preclinical animal studies	The interplay between vaccination and the activity of antimicrobials can be investigated Mechanistic understanding of the development of AMR	Laboratory experiments designed to address specific research questions	Inferring the findings to humans might be challenging and limits application of the results
Phase 1 and 2 studies with sample sizes of 100–200 participants	Mechanisms of vaccine impact on reducing AMR burden Effect of vaccination on the gut microbiome comparing vaccinated and unvaccinated individuals and by vaccine type Differences in ARG carriage between vaccinees and nonvaccinees at different timepoints following vaccination	Prospective longitudinal observation involving repeated sampling to assess the impact of vaccination on the AMR changes related to vaccination	Mostly addressed as an exploratory objective The small sample size limits validity and reliability Difficult to collect the required number of endpoints
Phase 3 studies—sample size of 300–3,000	Incidence of resistant infections from the pathogen of interest in vaccinees compared with nonvaccinees Antibiotic/antimicrobial use patterns of vaccinees compared with nonvaccinees Differences in ARG detection in gut microbiome between a subset of vaccinees and nonvaccinees	Prospective cohort studies with similar surveillance systems as the vaccine trial Genotyping and phylogenetic analysis for detection and quantification of genotypic resistance	Some of these objectives can be assessed as secondary endpoints with sample size estimation
Phase 4—cluster randomized and individually rando trials (RCT)	Direct, total, indirect, and overall impact of vaccines in reducing resistant infections. If addressed as a secondary endpoint with adequate statistical power, this could provide critical evidence for the impact of vaccines on reducing AMR Difference in AMU and AMC patterns between vaccinees and non-vaccinees; and/or vaccinated and unvaccinated clusters	In placebo-controlled vaccine RCTs, comparison between vaccinated and unvaccinated individuals and clusters can be performed In active controlled RCTs, the assessment for AMR could consider an unvaccinated control group Considerations for the impact of the active control is important	In the presence of an active control, assessment of impact might be complicated Understanding long-term impact might provide better-informed policy decisions
VE studies with no randomization	Incidence of resistant infections in vaccinees compared with nonvaccinees living in the same targeted community Estimate the overall reduction in resistant infections comparing before and after vaccination campaign periods in the specific region Trend in AMC patterns in the specific community after introduction of the specific vaccine compared with preintroduction levels	Case control studies comparing the impact of vaccines. Important to control for healthcare-seeking behavior by including hospital controls Test negative case control studies that provided comparable VE estimates to RCTs, for example, for typhoid [98] Prospective cohort studies employing active surveillance (at least enhanced passive surveillance) comparing vaccinated and unvaccinated groups	Representative surveillance established to detect endpoints are expensive and require expertise Differential healthcare-seeking behavior biases vaccine impact estimation Large sample sizes required to collect the required number of sample sizes
Government-led vaccine introduction	Assessing the overall reduction in resistant infections comparing pre- and postvaccination nationally Change in AMC patterns nationally after introduction of the specific vaccine as compared with preintroduction levels	Collecting and analyzing before- and after-vaccine-rollout data Representative sampling and follow up for 1 or more of AMR, AMU, or AMC	Data collection and analysis requires careful planning and implementation considering the vaccine introduction timelines Requires intensive planning and resources for data collection

Abbreviations: AMC, antimicrobial consumption; AMR, antimicrobial resistance; AMU, antimicrobial use; ARG, antimicrobial resistance gene; RCT, randomized controlled trial; VE, vaccine effectiveness.

## ECONOMIC BURDEN OF AMR

Data collected *a priori*, describing a significant reduction in AMU and AMR for primary and secondary infections following the WHO's AWaRe (Access, Watch, Reserve) guidelines for the classification of antibiotics [2], would enhance the economic case for vaccine introduction. Although evidence on the economic burden of AMR is currently lacking in many parts of the world, the economic burden was assessed as a global GDP loss ranging from 53 billion USD to >3 trillion USD, by 2050 (2011 USD values) [99]. In the United States, hidden costs from AMR specifically associated with ambulatory prescriptions for antibiotics, were estimated at 13 USD per prescription [100]. Data insufficiency, particularly in LMICs, hampers accurate calculations of AMR economic burden at the population level. Nonetheless, across 94 LMICs, costs of optimized immunization programs for 10 antigens (Hib, hepatitis B, human papillomavirus, Japanese encephalitis, measles, *Neisseria meningitidis* serogroup A, rotavirus, rubella, *S. pneumoniae*, and yellow fever) were projected at 34 billion USD between 2011 and 2020, offsetting illness costs of 586 billion USD and creating broader economic benefits of 1.5 trillion USD (2010 USD values) [101].

Robust calculations associated with AMR economic burdens at the population level require precise primary data points addressing disease-specific burdens and individual-level economic burdens for patients presenting for treatment because of AMR pathogens. For example, AMR to certain antibiotic classes in a patient may reduce the effectiveness of antibiotics for the current pathogen and, potentially, future infections [40, 41, 62, 82], worsening patient outcomes. This will particularly affect patients who make multiple visits to health facilities at varying times, requiring antibiotic treatment for different infections or who are exposed to resistant infections during hospitalization and intermediate or long-term care (ie, those recovering from surgical procedures).

## VACCINE HESITANCY

The use of vaccination to combat AMR mandates an urgent need to improve public perception of the value of vaccines. Vaccine hesitancy reached alarmingly high rates during the coronavirus disease 2019 (COVID-19) pandemic, and this may impact vaccination programs going forward [102]. Even in LMICs (where uptake has traditionally been better), by early 2022, uptake of the COVID-19 vaccine was less than 60% [103]. In many, this is accompanied by a paradoxical risk perception in terms of the safety of antibiotics compared with the potential dangers of vaccination [102, 104]. Accordingly, current vaccination programs—as well as future study enrollments as new vaccines are developed—are threatened. Patient education tools have previously enhanced the uptake of pneumococcal vaccines [105], and they may be a cost-effective way to improve the acceptance of vaccination.

## CONCLUSIONS AND FUTURE DIRECTIONS

Ongoing monitoring will establish long-term trends in the impact of vaccines. Strategies for the use of vaccines to combat emerging AMR must include better data collection and models for cost-effectiveness. Given the global platform to promote data collection on AMR and AMU/AMC [2] and the number of current and novel vaccines in the pipeline [106], there are excellent opportunities for funders and stakeholders to promote the inclusion of AMR impact studies as core to vaccine trials. AMR, particularly for the WHO-priority pathogens [2], should be inherent in any report on infectious disease burden and should be included in observational studies pre- and postvaccine introduction. Large-scale RCTs may provide more granular information but may be difficult to initiate for diseases with lower burdens or reduced carriage of pathogens. Better data are needed to understand differing side effects of certain vaccines in different vaccinees and why some vaccinees mount suboptimal immune responses. Stakeholders must capitalize on the use of social and other media to enhance dissemination of information, educate the public, and lobby politicians for support for antimicrobial stewardship and the value of vaccines. Reducing vaccine costs, improving immunogenicity, and facilitating attendance at vaccination centers may improve enrolment in vaccination programs. Furthermore, healthcare workers must promote vaccination over antimicrobials and provide knowledge and understanding to ensure patients receive appropriate messages. Ultimately, there are numerous arguments for improving how we quantify the impact of vaccines on AMR. We need to ensure that it is done.

## Supplementary Data

Supplementary materials are available at *Clinical Infectious Diseases* online. Consisting of data provided by the authors to benefit the reader, the posted materials are not copyedited and are the sole responsibility of the authors, so questions or comments should be addressed to the corresponding author.

## Supplementary Material

ciad562_Supplementary_Data

## Notes

***Disclaimer.*** The views expressed here are those of the authors and not necessarily those of the UK Department of Health and Social Care or its management agent, Mott MacDonald; and, International Vaccine Institute.

***Supplement sponsorship.*** This article appears as part of the supplement “Capturing Data on Antimicrobial Resistance Patterns and Trends in Use in Regions of Asia (CAPTURA),” funded by the Department of Health and Social Care's Fleming Fund using UK aid.
