# Supplementary material for: Vaccination to Reduce Antimicrobial Resistance Burden—Data Gaps and Future Research
Source: Clin Infect Dis. 2023 Dec 20;77(Suppl 7):S597–607. doi: 10.1093/cid/ciad562 (PMC10732565; doi:10.1093/cid/ciad562)
Supplement: ciad562_Supplementary_Data [file ciad562_supplementary_data.zip › Supplementary Table S1.docx]

**Supplementary Table S1**. Clinical vaccine trials in clinicaltrials.org and their AMR objectives

| **№** | **Disease** | **Vaccine** | **AMR objective and associated outcome** | **Participant population** | **Age** | | **Number of participants** | | **Recruitment Status** | **Clinical trial phase** | **Clinical trial ID** |
| --- | --- | --- | --- | --- | --- | --- | --- | --- | --- | --- | --- |
|  |  |  |  |  | *Adult* | *Child* | *Male*  *Participants* | *Female*  *Participants* |  |  |  |
| 1 | Gonorrhea Infection in Gay and Bisexual Men | Meningococcal B 4CMenB (Bexsero®)  2 dose | *Secondary outcome*  Strain specific (by whole genome sequence or **antimicrobial resistance** phenotype) - first instance of the detection of N. gonorrhoeae infection in a urine sample or on a swab taken from the urethra, anorectum, oropharynx, or vagina at a study visit. | Men (cis and trans), trans women, and non-binary people who had sex with at least one man in the preceding 6 months | 18–50 years old |  | 730 (still recruiting) |  | Recruiting | Phase 3  Interventional | [NCT04415424](https://clinicaltrials.gov/ct2/show/NCT04415424?term=antimicrobial+resistance&cond=vaccine&draw=2&rank=3) |
| 2 | Nasopharyngeal Carriage | Pneumococcal conjugate vaccines (PCVs) | *Secondary outcome*  Detect the emergence of resistant *S. pneumoniae.*  For the ancillary study, the resistance of E. coli (ESBL) will be evaluated. |  |  | 6  months to 24 months for AOM.  6 months to 15 years for healthy children | All sexes  23560 | | Recruiting | Interventional | [NCT04460313](https://clinicaltrials.gov/ct2/show/NCT04460313?term=antimicrobial+resistance&cond=vaccine&draw=2&rank=5) |
| 3 | Chronic Hepatitis B | HBV DNA vaccine | The different occurrence rates of HBV Drug Resistance Gene (YMDD) between the 2 study arms | Chronic Hepatitis B patients: HBeAg-positive | 18 Years to 65 Years |  | All sexes  33 | | Unknown  (In 2020: Active, not recruiting) | Phase 2 | [NCT01189656](https://clinicaltrials.gov/ct2/show/NCT01189656) |
| 4 | Pneumococcal Infections | Pneumococcal conjugate vaccine (PCV) | Rate of antibiotic prescription at outpatient care among children aged below 5 years of age. Time Frame: 24 months, relative to baseline (pre-vaccination). | Children in a camp for displaced persons outside Hargeisa, Somaliland |  | 6 weeks to 14 years | N/A (1500 estimated) | | Not yet recruiting | Phase 4 | [NCT04945681](https://clinicaltrials.gov/ct2/show/NCT04945681) |
| 5 | Streptococcus Pneumoniae | Seven-valent pneumococcal conjugate vaccine (7v-PCV) - Prevenar | *Secondary Outcome*  Decrease infections related to *S. pneumoniae* in infants (and indirectly elderly of same family) and decrease use of antibiotics and therefore resistance. | Infants who were enrolled in the time period May 18th, 2009, to August 10th, 2010, would have the 7v PCV |  | 2 months to 2 years | All sexes  300 | | Unknown (last update posted: 2009) | Phase 1  Phase 2  Interventional | [NCT00900978](https://clinicaltrials.gov/ct2/show/record/NCT00900978?term=resistance&cond=vaccine&intr=vaccine&titles=vaccine&outc=antimicrobial+resistance&draw=2&rank=2) |
| 6 | Community Acquired Pneumonia (CAP) | 13-valent Pneumococcal Conjugate Vaccine (PCV13) | *Secondary outcome*  Frequency of antibiotic resistance among *S. pneumoniae* isolates by type. Proportion of *S. pneumoniae* isolates with antibiotic resistance identified by standard of care testing, overall, and by resistance type | Adults aged ≥60 years in the Region of Madrid (Spain) | 60 years and over |  | All sexes  9000 | | Recruiting | Observational | [NCT04613375](https://clinicaltrials.gov/ct2/show/study/NCT04613375?term=resistance&cond=vaccine&intr=vaccine&titles=vaccine&outc=antimicrobial+resistance&draw=2&rank=5) |
| 7 | Malaria | Arm 1: RTS,S/AS01 Malaria Vaccine  Arm 2: tetanus/diphtheria toxoids vaccine | *Secondary outcomes*  Drug resistance to SP and AQ: The presence of molecular markers of resistance to SP and AQ in parasite positive samples  SP+AQ drug sensitivity: The 28-day treatment outcome in children with asymptomatic malaria parasitemia treated with SP+AQ | Children under 5 years living in areas of Burkina Faso or Mali with intense malaria transmission |  | 3 to 5 years | 5045 | | Completed | Phase 3 | [NCT04319380](https://clinicaltrials.gov/ct2/show/NCT04319380) |
| 8 | Infections, Streptococcal | Biological: Pneumococcal conjugate vaccine GSK1024850A  Biological: Havrix  Biological: Engerix-B  Biological: Infanrix hexa  Biological: GSK Biologicals' DTPa-IPV/Hib vaccine | *Secondary outcome*  Number of subjects with any antibiotic prescription at least once during the entire study period, in the carriage subset | Healthy children less than 3 years old. Between, and including, 6 and 16 weeks of age at the time of the first vaccination living in the area covered by the surveillance system for community acquired pneumonia (CAP), invasive disease, and acute otitis media (AOM) |  | Children less than 3 years old | Total: 23802  AMU objective for subgroup of 2,000 subjects | | Completed | Phase 3 | [NCT00466947](https://clinicaltrials.gov/ct2/show/NCT00466947) |
| 9 | Influenza | Northern Hemisphere Influenza Vaccine 2017/18 | *Secondary outcome*  Health Care Utilization: Via medical records, they assessed health care usage potentially attributable to flu-like symptoms (e.g., GP visits, hospitalization, antibiotic prescription) during the 6 months post-vaccination | Males and Females aged 65–85 years (inclusive) who received influenza vaccination for the 2016/17 season | 65 Years to 85 Years |  | 106 | | Completed | N/A | [NCT03144518](https://clinicaltrials.gov/ct2/show/NCT03144518) |
| 10 | Physician-diagnosed otitis media (OM) and acute otitis media (AOM) | Pneumococcal conjugate vaccines (PCV) | *Secondary outcomes*  Assessment of the impact of PCVs by describing trends over time in the rate of antibiotics dispensed for OM and AOM diagnoses among children with OM or AOM.  Assessment of the Costs for OM- and AOM-related healthcare resource utilization | Swedish children living in the County councils of VGR and Skåne |  | Children ≤5 years old | N/A - Retrospective review of medical records | | Completed | N/A  [Restrospective study](https://pubmed.ncbi.nlm.nih.gov/32574101/) | [NCT02742753](https://clinicaltrials.gov/ct2/show/NCT02742753) |
| 11 | Chronic Obstructive Pulmonary Disease  Asthma  Pneumococcal Infections | Biological: Prevenar-13 (Conjugate 13 serotype pneumococcal vaccine)  Biological: Pneumo-23 (Polysaccharide 23-valent pneumococcal vaccine) | *Primary outcomes*  Number of patients without exacerbations of the underlying disease, antibiotic use, and hospitalization  The number of exacerbations of the underlying disease, antibiotic use, and hospitalization | Adult patients with bronchopulmonary pathology | 18 Years to 80 Years |  | 219 | | Completed | Phase 4 | [NCT02787863](https://clinicaltrials.gov/ct2/show/NCT02787863) |
| 12 | Influenza | Quadrivalent inactivated influenza vaccine (QIV)  2019-2020 QIV Vaxigrip-Tetra | *Secondary outcome*  Rates of acute respiratory infection, influenza-like illness, febrile episode, use of antibiotics, use of antivirals, pneumonia, acute otitis media (for infants only), healthcare seeking and hospitalization in pregnant women and their infants | Pregnant women and their infants | 18 Years to 45 Years (Adult) |  | 949 TOTAL  636 pregnant women and 474 infants | | Completed | Observational | [NCT04723771](https://clinicaltrials.gov/ct2/show/NCT04723771) |
| 13 | Non-specific Effects of Vaccines  Respiratory illness, Covid19 | *Mycobacterium bovis* BCG live attenuated BCG-Denmark vaccine | *Primary outcome*  Acute infection identified either by a doctor, antibiotics use, hospitalization, or death due to infection. | Senior citizens | 65 Years to 110 Years |  | 1700 | | Active, not recruiting | Phase 3 | [NCT04542330](https://clinicaltrials.gov/ct2/show/NCT04542330) |
| 14 | Bronchospasm; Bronchiolitis  Bronchospasm; Bronchitis | Bacterial vaccine | *Secondary outcomes*  Medication Score During WA: Review of medication consumption during wheezing attacks. In the case of antibiotics and antipyretic/-anti-inflammatory drugs, the number of daily doses of these drugs was recorded.  Overall Medication Score: Review of medication consumption during the whole study. The patients were reviewed at our clinics every three months. Medication scores were recorded daily in a diary card by the parents, who were duly instructed. | Children with recurrent bronchospasms (wheezing attacks); 3 or more exacerbations in the last 12 months |  | 12 to 36 months | 120 | | Completed | Phase 3 | [NCT01734811](https://clinicaltrials.gov/ct2/show/NCT01734811) |
| 15 | Chronic Obstructive Pulmonary Disease | 13-valent pneumococcal conjugated vaccine (PCV-13) | *Secondary outcome*  Any carriage isolates of *S. pneumoniae* isolated from COPD patients are assessed for serotype and antibiotic resistance profiles to determine if they are covered by current pneumococcal vaccines. | Adult patients suffering from chronic obstructive pulmonary disease (COPD) | 18 years and older |  | 150 | | Completed | Observational | [NCT02535546](https://www.clinicaltrials.gov/ct2/show/study/NCT02535546?intr=vaccine&outc=antimicrobial+usage+OR+antimicrobial+consumption+OR+antibiotic+prescription&draw=3&rank=18) |
| 26 | Tuberculosis (TB) | Bacille Calmette Guérin (BCG) | *Secondary Outcome*  To test that infants who get the BCG vaccine at birth are prescribed less antibiotics during early childhood than non-BCG-immunized infants. Use of antibiotics was defined as one or more prescriptions of systemic antibiotics. | Infants born at Rigshospitalet, Hvidovre Hospital and Kolding Hospital before gestational age 32 weeks and/or birth weight <1,000 g. |  | Up to 7 Days | 4262 | | Completed | Interventional  Phase 4 | [NCT01694108](https://www.clinicaltrials.gov/ct2/show/study/NCT01694108?intr=vaccine&outc=antimicrobial+usage+OR+antimicrobial+consumption+OR+antibiotic+prescription&draw=3&rank=11) |
