# Supplementary figures and images for: Vaccination to Reduce Antimicrobial Resistance Burden—Data Gaps and Future Research
Source: Clin Infect Dis. 2023 Dec 20;77(Suppl 7):S597–607. doi: 10.1093/cid/ciad562 (PMC10732565; doi:10.1093/cid/ciad562)

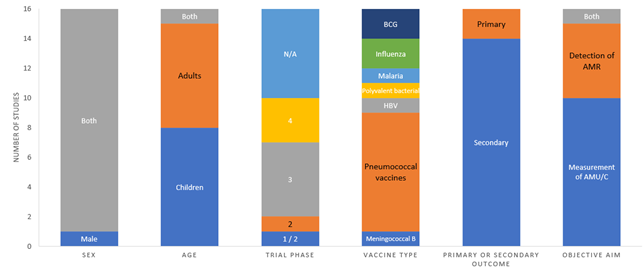

Supplement: ciad562_Supplementary_Data [file ciad562_supplementary_data.zip › Figure S1.tif]
